# Significant Prevalence of Dual KPC/NDM Carbapenemase-Producing *Klebsiella pneumoniae* in an ICU Cohort in Thessaloniki (2023), Including an ST512 Isolate Co-Harboring *bla_NDM-1_* and *bla_KPC-3_*

**DOI:** 10.3390/antibiotics14100994

**Published:** 2025-10-04

**Authors:** Maria Chatzidimitriou, Apostolos Voulgaridis, Pandora Tsolakidou, Fani Chatzopoulou, Ioannis Chonianakis, Eleni Vagdatli, Melania Kachrimanidou, Timoleon-Achilleas Vyzantiadis

**Affiliations:** 1 Department of Biomedical Sciences, Faculty of Health Sciences, International Hellenic University, 57400 Thessaloniki, Greece; fchatzopoulou@ihu.gr (F.C.); chonianakis2000@gmail.com (I.C.); 2 Department of Microbiology, Hippokration General Hospital, 54642 Thessaloniki, Greece; apovoulga1987@gmail.com (A.V.); evagdatli@gmail.com (E.V.); 3 Department of Microbiology, General Hospital of Volos, 38222 Volos, Greece; ptsolakidou@gmail.com; 4 Department of Microbiology, Medical School, Aristotle University of Thessaloniki, 54124 Thessaloniki, Greece; melinaka@auth.gr (M.K.); avyz@med.auth.gr (T.-A.V.)

**Keywords:** *Klebsiella pneumoniae*, carbapenem resistance, *bla_KPC_*, *bla_NDM_*, multidrug resistance (MDR), antimicrobial susceptibility, Intensive Care Unit (ICU) infections, Greece

## Abstract

**Background/Objectives**: Carbapenem-resistant *Klebsiella pneumoniae* (CRKP) threatens Intensive Care Units (ICU), particularly in settings where serine (KPC) and metallo-β-lactamases (NDM) co-circulate. The aim of this study was to assess CRKP susceptibility especially to novel β-lactam/β-lactamase inhibitor combinations, characterize the genetic determinants of resistance, and contribute to the understanding of local epidemiology in the ICU of our hospital. **Methods**: We studied 32 non-duplicate CRKP isolates (30 ICU, 2 wards) collected at Hippokration General Hospital, Thessaloniki (May–Oct 2023). Bacterial identification and Antimicrobial susceptibility testing (AST) were performed by VITEK-2; Minimum inhibitory concentrations (MICs) for ceftazidime/avibactam (CAZ/AVI), meropenem/vaborbactam (MER/VAB), and imipenem/relebactam (IMI/REL) were determined by E-tests. Colistin MICs were performed by broth microdilution. Carbapenemases were screened phenotypically and by immunochromatography and confirmed by multiplex PCR. One bronchial isolate co-harboring *bla*_NDM_ and *bla*_KPC_ genes underwent WGS. **Results**: All isolates were carbapenem-resistant and showed extensive resistance to β-lactams and fluoroquinolones. By PCR, 8/32 (25%) carried *bla_KPC_* alone, 8/32 (25.0%) *bla_NDM_* alone, and 16/32 (50%) co-harbored *bla_KPC_* and *bla_NDM_*. KPC-only isolates were generally susceptible in vitro to CAZ/AVI, MER/VAB, and IMI/REL, whereas dual KPC-NDM producers were resistant to all three combinations. Tigecycline showed the highest retained activity; colistin remained active in a minority. WGS of one ST512 (CG258) isolate revealed co-harboring *bla_NDM-1_* and *bla_KPC-3_* with additional resistance determinants and plasmid replicons, consistent with high-risk spread. **Conclusions**: Half of CRKP isolates in this ICU-predominant series co-produced KPC and NDM, severely limiting β-lactam/β-lactamase inhibitor options. These data support routine screening for carbapenemases, strict infection prevention, antimicrobial stewardship, and access to agents active against MBLs.

## 1. Introduction

Carbapenem-resistant *Klebsiella pneumoniae* (CRKP) has emerged as one of the most critical challenges in modern intensive care medicine. The World Health Organization (WHO) has designated CRKP as a top-priority pathogen for the development of new antimicrobials due to its rapid global dissemination and association with multidrug resistance [1]. Outbreaks in intensive care units (ICUs) across Europe, Asia, and the Americas have been linked to excess mortality rates approaching 40–50%, prolonged hospitalization, and substantially increased healthcare costs [2,3].

The emergence of high-risk international clones—such as ST258, ST11, ST15, and ST512—further underscores the ability of CRKP to spread across continents through both clonal expansion and horizontal gene transfer [4,5,6].

In ICUs, the burden of CRKP is amplified by the unique vulnerability of patients. These individuals are often immunocompromised, exposed to invasive devices, and subjected to prolonged courses of broad-spectrum antibiotics, all of which accelerate selective pressure and foster the emergence of extensively drug-resistant strains [7]. Consequently, infections caused by *K. pneumoniae*—including urinary tract infections, ventilator-associated pneumonia (VAP), bacteremia, and surgical site infections—are associated with worse clinical outcomes compared to infections by susceptible strains [8].

The resistance of *K. pneumoniae* is largely mediated by the production of β-lactamases, particularly extended-spectrum β-lactamases (ESBLs) and carbapenemases such as *K. pneumoniae* carbapenemase (KPC), New Delhi metallo-β-lactamase (NDM), and Verona integron-encoded metallo-β-lactamase (VIM) [9]. These enzymes not only compromise the efficacy of carbapenems but also undermine the effectiveness of novel β-lactam/β-lactamase inhibitor combinations. While agents such as ceftazidime/avibactam and meropenem/vaborbactam have been introduced to target multidrug-resistant strains [10,11], clinical resistance has already been reported [12], raising concerns about the sustainability of current therapeutic options and underscoring the need for alternative approaches, including phage therapy and biofilm inhibitors [13].

Greece has been at the epicenter of CRKP spread in Europe for more than a decade, with KPC producers dominating earlier epidemics and NDM producers now increasingly detected in several tertiary care hospitals [14,15]. Previous studies from Hippokration General Hospital of Thessaloniki identified *K. pneumoniae* strains co-producing KPC and VIM, as well as emerging ST11, ST15, and ST512 isolates harboring *bla*_NDM-1_ [16,17]. Building on this context, the present study analyzed 32 CRKP isolates collected in 2023, primarily from ICU patients. The objectives were to assess their susceptibility to novel β-lactam/β-lactamase inhibitor combinations, characterize the genetic determinants of resistance, and contribute to the understanding of local ICU epidemiology in Thessaloniki.

## 2. Results

### 2.1. Demographics

Between May and October 2023, 32 carbapenem-resistant *K. pneumoniae* isolates were recovered from hospitalized patients at Hippokration General Hospital. Most originated from ICU (30/32; 93.8%), while two were from medical wards. Most isolates were obtained from bronchial secretions (34.3%), followed by blood cultures (18.7%) central venous catheters (15.6%), urine (12.5%), drainage fluids (9.3%), sputum (6%), wound (3.1%) and cerebrospinal fluid (3.1%) (Table 1).

### 2.2. Antimicrobial Susceptibility

Antibiotic susceptibility testing with the VITEK-2 system confirmed that all isolates were resistant to carbapenems and fluoroquinolones. Susceptibility was observed for ceftazidime/avibactam (25%) imipenem/relebactam (25%), meropenem/vaborbactam (25%), gentamicin (28.1%), tigecycline (87.5%), colistin (37.5%), and trimethoprim/sulfamethoxazole (12.5%) (Table 1, Table 2 and Table 3).

### 2.3. Phenotypic Detection of Carbapenemases

Phenotypic synergy testing and immunochromatography identified the production of KPC and/or NDM carbapenemases in all isolates.

### 2.4. Molecular Assays

Multiplex PCR confirmed the majority (90%) of phenotypic and immunochromatographic results. Every isolate harbored at least one gene, with *bla_KPC_* (8) and *bla_NDM_* (8), and sixteen isolates co-harbored both genes.

The D4370 isolate, identified as *K. pneumoniae* ST512 (GRTHES), harbored both *bla_NDM-1_* and *bla_KPC-3_* genes. It exhibited resistance to all β-lactams (including ceftazidime/avibactam, meropenem/vaborbactam, and imipenem/relebactam), aminoglycosides, fluoroquinolones, fosfomycin, trimethoprim–sulfamethoxazole and colistin, with tigecycline as the only remaining effective agent (Table 2). Genomic analysis revealed multiple resistance determinants (e.g., *bla_TEM-1_*, *bla_CTX-M-15_*, *oqx*A/*oqx*B, *fos*A), point mutations (*gyr*A S83I, *par*C S80I, *mgr*B G37S, *pmr*B R256G, *omp*K36_D135DGD), and plasmid replicons (IncFIA(HI1), IncFIB(K), IncFII(K), IncX3) that promote horizontal gene transfer. The isolate also carried virulence factors such as yersiniabactin siderophore genes and adhesion proteins, underscoring its pathogenic potential [18]. Key findings of the next generation sequencing are summarized in Table 4.

## 3. Discussion

Globally carbapenem resistance in *K. pneumoniae* in healthcare systems is mainly mediated by the production of carbapenemases, such as KPC and MBLs (NDM, VIM). The widespread presence of genes encoding these enzymes, often located on mobile genetic elements, facilitates clonal expansion and interstrain dissemination [19,20,21,22].

A multicenter survey across Southern Europe demonstrated significant geographical variability in carbapenemase profiles, with *bla_KPC_* predominating in Greece, Italy, and Spain, while *bla_OXA-48_*, was more frequent in Serbia and Romania [23]. More recently, an increase in *bla_NDM-1_* -producing isolates has been reported in Greece, signaling the expansion of a widespread clonal outbreak [24].

Previous studies from Greek hospitals have documented the dominance of ST258 and other high-risk sequence types such as ST39, ST11, ST147, and ST15, all of which have been implicated in epidemic spread and nosocomial outbreaks [25,26,27].

In our study, antimicrobial susceptibility testing (Table 3 and Table 4) showed that all isolates were resistant to older β-lactam antibiotics, with the exception of a single *bla_NDM_* positive isolate that remained susceptible to aztreonam. All eight isolates carrying only the *bla_KPC_* gene were susceptible in vitro to the newer β-lactam/β-lactamase inhibitor combinations-ceftazidime/avibactam, meropenem/vaborbactam, and imipenem/relebactam. Among aminoglycosides, only gentamicin retained activity (28.1%), although its clinical use is limited by nephrotoxicity and it is generally reserved for combination therapy, often alongside β-lactams. Tigecycline demonstrated in vitro activity against the majority of isolates (87.5%), but its approved indications by the FDA are limited to intra-abdominal and skin/soft-tissue infections, despite some evidence supporting its use in bloodstream infections [28]. Colistin remained active against 37.5% of isolates, including 5 of the 15 co-harboring *bla_NDM_* and *bla_KPC_*. However, its therapeutic value is constrained by nephrotoxicity and the risk of resistance development through mutations affecting lipid membrane synthesis, necessitating its use in combination regimens.

Multiplex PCR analysis in our study revealed the presence of *bla_KPC_* in 25% of isolates, *bla_NDM_* in 25.0%, and both genes simultaneously in 50%. The frequent co-production of *bla_KPC_* and *bla_NDM_* underscores the significant therapeutic challenges posed by these strains, as it undermines the efficacy of last-line agents.

The therapeutic implications of these findings are considerable. While ceftazidime/avibactam retains activity against KPC-producing strains, it is ineffective against NDM and thus loses utility in isolates co-harboring both enzymes. Other novel agents, including imipenem/relebactam and meropenem/vaborbactam, are also valuable options against KPC producers but are similarly compromised in the presence of dual carbapenemases, as these inhibitors lack activity against metallo-β-lactamases. In such cases, treatment often relies on combination regimens, since newer agents with activity against MBLs, such as cefiderocol and aztreonam/avibactam, are not yet available in Greece.

Whole-genome analysis of the D4370 (GRTHES) ST512 strain underscores the clinical importance of dual carbapenemase-producing *K. pneumoniae*. ST512, a high-risk clone within the CG258 clonal group, has previously been associated with *bla_KPC_* in Greece [29]; however, the concurrent acquisition of *bla_NDM-1_* represents a significant epidemiological shift. Of particular interest is the detection of the *bla_KPC-3_* gene in this isolate, which we first reported in 2024 as part of a co-producing NDM-1 and KPC-3 *K. pneumoniae* ST512 strain recovered from a bronchial secretion in an ICU patient in Greece [18]. Comparable findings have been documented in other countries, underscoring that the emergence of dual carbapenemase producers is a regional phenomenon rather than an isolated occurrence. In Italy, KPC-3/NDM-1 co-producing *K. pneumoniae* have been reported in bloodstream infections, while OXA-48/NDM-1 combinations were recently described in two hospitals in southern Italy [30,31]. In Turkey, both KPC/NDM and OXA-48/KPC co-producers have been detected, including ST307 isolates from emergency department cases [32,33]. Romania has reported KPC-2/VIM-1 producers as early as 2015 and, more recently, NDM/OXA-48-like combinations in ICU settings associated with high mortality [34,35]. In China NDM-5 has been increasingly identified, frequently together with OXA-48-like carbapenemases in ST11 and ST15 isolates [36,37]. Collectively, these data highlight a worrisome regional trend towards the circulation of *K. pneumoniae* harboring multiple carbapenemases, which severely compromises therapeutic options and increases the risk of cross-border dissemination. Further studies are needed to determine whether this represents a single event or the beginning of broader dissemination. The identification of multiple plasmids, integrative conjugative elements, and insertion sequences highlights the potential for horizontal gene transfer, while the co-resistance to fluoroquinolones, aminoglycosides, and colistin severely limits available therapeutic options.

In this ICU-predominant cohort, nearly 50% of CRKP co-produced KPC and NDM, a combination that abrogates the activity of currently available serine-targeted BL/BLI regimens (CAZ/AVI, MER/VAB, IMI/REL). Tigecycline and, less consistently, colistin remained options but with important PK/PD and toxicity constraints. The ST512 (CG258) genome co-harboring blaNDM-1 and blaKPC-3 illustrates ongoing convergence of high-risk clones with multiple mobile genetic elements, underscoring the need for rapid carbapenemase genotyping, reinforced infection prevention, and improved access to agents active against MBLs.

### Limitations of the Study

This study has several limitations. The relatively small number of isolates restricts the ability to draw firm conclusions regarding the local epidemiology of the ICU at Hippokrateion Hospital. Multilocus sequence typing (MLST) or whole-genome sequencing (WGS) could not be performed for all isolates because of limited funding. The available clinical data were also incomplete, as information on the duration of ICU stay before sampling, the underlying reason for ICU admission, and whether the isolates represented true infection or colonization was not consistently documented. In addition, data on prior antimicrobial exposure within the preceding three months were unavailable. Although antimicrobial susceptibility testing was conducted using standardized protocols (VITEK 2 and broth microdilution), internal quality control strains were not systematically included in all runs, which may have influenced the precision of MIC determinations. Finally, MIC_50_ and MIC_90_ values were not calculated due to the limited sample size, and future studies with larger isolate collections are needed to provide these distributional measures.

## 4. Materials and Methods

Phenotypic tests were conducted in the Microbiology Department of Hippokration General Hospital of Thessaloniki between May and October 2023, following approval from the laboratory director, departmental supervisors, the hospital administration, and the Bioethics Committee of Aristotle University of Thessaloniki No 5/12.03.2024.

### 4.1. Study Setting and Sample Collection

A total of 32 carbapenem-resistant *K. pneumoniae* isolates were obtained from clinical specimens of hospitalized patients at Hippokration General Hospital. Of these, 30 were recovered from ICU patients and 2 from medical wards (Departments 1 and 4). Clinical specimens included bronchial secretions (*n* = 11), blood cultures (*n* = 6), central venous catheters (*n* = 5), urine (*n* = 4), drainage fluids (*n* = 2), sputum (*n* = 2), wound swab (*n* = 1), and cerebrospinal fluid (*n* = 1). Isolates were selected based on resistance to at least one carbapenem (imipenem, meropenem, or ertapenem) and were stored at −80 °C until analysis.

### 4.2. Bacterial Identification and Susceptibility Testing

Specimens were cultured on standard media, including blood agar, MacConkey agar, and Mueller–Hinton agar (OXOID Ltd., Hampshire, UK). After 24 h of incubation, bacterial identification and initial antimicrobial susceptibility testing were performed using the VITEK-2 automated system (bioMérieux, Marcy-l’Étoile, France) using three Gram-negative panels (AST-N233, AST-N426, XN26).

Susceptibility to novel antibiotic combinations—imipenem/relebactam and meropenem/vaborbactam—was further determined using E-test minimum inhibitory concentration (MIC) assays (Liofilchem, Roseto degli Abruzzi, Italy). Colistin susceptibility was assessed by broth microdilution in cation-adjusted Mueller–Hinton broth (CAMHB, Liofilchem, Roseto degli Abruzzi, Italy). Breakpoints were interpreted according to the European Committee on Antimicrobial Susceptibility Testing (EUCAST, version 13.0, 2023) guidelines (accessed on 1 December 2023) [38], while tigecycline susceptibility was interpreted using U.S. Food and Drug Administration (FDA) criteria (susceptible ≤ 2 µg/mL; intermediate = 4 µg/mL; resistant ≥ 8 µg/mL). For descriptive purposes, intermediate and susceptible isolates were grouped together (S + I). Carbapenem resistance was defined as resistance to at least one carbapenem.

### 4.3. Phenotypic Screening for Carbapenemase Activity

Phenotypic detection of carbapenemase production was performed using the synergy method with meropenem disks (OXOID Ltd., Hampshire, UK) in combination with ethylenediaminetetraacetic acid (EDTA; Sigma-Aldrich, St. Louis, MO, USA) and phenylboronic acid (Sigma-Aldrich, St. Louis, MO, USA), targeting metallo-β-lactamases (MBLs) and KPC enzymes, respectively.

Carbapenemase identification was further processed with a multiplex lateral flow immunoassay (NG-Test CARBA 5, NG Biotech, Guipry-Messac, France). The assay detection limits were 150 pg/mL for NDM, 600 pg/mL for KPC, 200 pg/mL for IMP, 300 pg/mL for VIM, and 300 pg/mL for OXA-48-like enzyme.

### 4.4. Molecular Assays Results

#### 4.4.1. Multiplex PCR

Results were confirmed by multiplex PCR which was performed in the Biomedical Sciences’ Laboratory of the International Hellenic University of Greece.

From an overnight blood agar culture, 3–4 colonies were collected with a sterile loop and suspended in 100 μL of distilled water, followed by vortex mixing. Cell lysis was performed at 95 °C for 10 min, and cellular debris was removed by centrifugation at 14,000 rpm for 3 min. Two microliters of the resulting supernatant were used as the DNA template in a 50 μL multiplex PCR reaction targeting the *bla_KPC_*, *bla_NDM_*, *bla_OXA-48_*, and *bla_VIM_* genes. The primers used for amplification (Table 5) were previously described [19,20]. The expected amplicon sizes were 438 bp for *bla_OXA-48_*, 621 bp for *bla_NDM_*, 798 bp for *bla_KPC_*, and 390 bp for *bla_VIM_*.

PCR amplification was carried out using 2 U of AmpliTaq Gold DNA Polymerase (Thermo Fisher Scientific Inc., Applied Biosystems, Waltham, MA, USA) and 0.3 μmol/L of each primer, following the manufacturer’s recommendations. The cycling conditions consisted of an initial denaturation at 94 °C for 10 min; 36 cycles of denaturation at 94 °C for 30 s, annealing at 52 °C for 40 s, and extension at 72 °C for 50 s; and a final extension at 72 °C for 6 min. PCR products were resolved by electrophoresis on a 2% agarose gel containing 0.5 μg/mL ethidium bromid. Primers used are shown in Table 5.

#### 4.4.2. Whole-Genome Sequencing

A single isolate, with laboratory I.D D4370 (designated as GRTHES for publication purposes), recovered from the bronchial secretions of an ICU patient in August 2023, was subjected to whole-genome sequencing (WGS) for in-depth characterization [10]. This isolate was selected because it co-harbored both *bla_KPC_* and *bla_NDM_* genes as a representative between other isolates. Whole-genome sequencing (WGS) was carried out in a private laboratory in Greece. Library preparation was performed using Ion Torrent technology and the Ion Chef Flow Diagram (Thermo Fisher Scientific, Waltham, MA, USA). Sequencing was conducted on the Ion 5SXLS system, and raw data were processed with Ion Torrent Suite v.s1010 (Thermo Fisher Scientific, Waltham, MA, USA). Subsequent analyses were performed using the Galaxy Server platform and the Centre for Genomic Epidemiology database.

Read quality was assessed with FastQC (Galaxy version 0.75+Galaxy0), and quality improvement was achieved with the FASTQ Quality Trimmer using a sliding window approach (Galaxy version 1.1.5). Genome assembly was carried out using the Unicycler pipeline within the Create Assemblies tool (Galaxy version 0.5.0+Galaxy0). Resistance and virulence genes were screened with ABRicate (Galaxy version 1.0.1), while plasmid replicons were identified using PlasmidFinder (Galaxy version 2.1.6+Galaxy1). Capsular (K locus) and O-antigen serotypes were determined using Kaptive (https://kaptive-web.erc.monash.edu/, accessed on 10 November 2024). Integrative conjugative elements (ICEs) were identified with ICEberg 3.0 (https://tool2-mml.sjtu.edu.cn/ICEberg3/ICEfinder.php, assessed on 10 November of 2024).

## 5. Conclusions

Dual KPC/NDM producers accounted for nearly half of carbapenem-resistant *K. pneumoniae* isolates in this ICU-predominant cohort, substantially eroding the utility of currently available β-lactam/β-lactamase inhibitor regimens active only against serine carbapenemases. Tigecycline retained the highest in vitro activity, and colistin remained active in a minority; however, toxicity and PK/PD limitations constrain both agents.

The ST512 (CG258) genome co-harboring *bla_NDM-1_* and *bla_KPC-3_*, along with multiple resistance determinants and plasmid replicons, underscores the capacity for horizontal spread and treatment failure risk. These findings support rapid carbapenemase genotyping, strict infection-prevention measures, and stewardship, and argue for access to agents with anti-MBL activity (e.g., Aztreonam/avibactam or Cefiderocol) in Greece.

## Figures and Tables

**Table 1:** Isolate metadata (Laboratory ID, specimen, department, date, β-lactamase genes, antibiotic susceptibility. Abbreviations: I: Susceptible, increased exposure (Eucast), I: Intermediate (F.D.A).

Laboratory ID	Specimen	Department	Date Collected	β-Lactamase Genes (Multiplex PCR)	Antibiotic Susceptibility (S + I)
A9072	blood	ICU	16 May 2023	*bla_KPC_*	Ceftazidime/avibactam, Meropenem/vaborbactam, Imipenem/relebactam, Gentamicin
A11159	blood	ICU	15 May 2023	*bla_KPC_*	Ceftazidime/avibactam, Meropenem/vaborbactam, Imipenem/relebactam, Gentamicin, Trimethoprim/sulfamethoxazole, Tigecycline, Fosfomycin, Colistin
A11694	blood	ICU	22 June 2023	*bla_NDM_*	Tigecycline
A15330	blood	ICU	9 August 2023	*bla_KPC_*	Ceftazidime/avibactam, Meropenem/vaborbactam, Imipenem/relebactam, gentamicin, Trimethoprim/sulfamethoxazole, Intermediate (I) to Tigecycline
A18251	blood	ICU	9 September 2023	*bla_KPC_*, *bla_NDM_*	Tigecycline (I)
A20170	blood	ICU	17 October 2023	*bla_NDM_*	Colistin
B7232	urine	4th Internal Department	9 May 2023	*bla_NDM_*	Colistin
B9217	urine	ICU	14 June 2023	*bla_NDM_*	Colistin, Tigecyclin, Trimethoprim/sulfamethoxazole
B12743	urine	ICU	16 August 2023	*bla_NDM_*	Aztreonam (I), Tigecycline (I), Levofloxacin (I)
B13435	urine	ICU	28 August 23	*bla_KPC_*, *bla_NDM_*	Tigecycline
C4171	drainage	ICU	14 June 2023	*bla_NDM_*	Aztreonam, Gentamicin, Tigecycline
C5596	drainage	ICU	9 August 2023	*bla_KPC_*	Ceftazidime/avibactam, Meropenem/vaborbactam, Imipenem/relebactam, Gentamicin, Tigecycline (I)
C6168-1	wound	ICU	1 September 23	*bla_KPC_*, *bla_NDM_*	Tigecycline
D2415	cerebrospinal fluid	ICU	9 May 2023	*bla_KPC_*, *bla_NDM_*	Colistin, Tigecycline
D2606	bronchial secretions	ICU	18 May 2023	*bla_KPC_*	Ceftazidime/avibactam, Meropenem/vaborbactam, Imipenem/relebactam, Gentamicin, Colistin, Tigecycline
D2678	sputum	1st Internal Department	22 May 2023	*bla_KPC_*, *bla_NDM_*	Gentamicin, Colistin, Tigecycline
D2803	bronchial secretions	ICU	31 May 2023	*bla_KPC_*, *bla_NDM_*	Colistin, Tigecycline
D2845	bronchial secretions	ICU	2 June 2023	*bla_KPC_*, *bla_NDM_*	Tigecycline
D3437-2	bronchial secretions	ICU	30 June 2023	*bla_KPC_*	Ceftazidime/avibactam, Meropenem/vaborbactam, Imipenem/relebactam, Gentamicin, Colistin, Tigecycline
D4109	sputum	ICU	8 August 2023	*bla_KPC_*, *bla_NDM_*	Tigecycline
D4140	bronchial secretions	ICU	10 August 2023	*bla_KPC_*	Gentamicin, Tigecycline (I), Trimethoprim/sulfamethoxazole
D4175	bronchial secretions	ICU	14 August 2023	*bla_KPC_*, *bla_NDM_*	Tigecycline
D4370	bronchial secretions	ICU	2 August 2023	*bla_KPC_*, *bla_NDM_*	Colistin, Tigecycline
D4449	bronchial secretions	ICU	30 August 2023	*bla_NDM,_*, *bla_KPC_*	Tigecycline
D4459	bronchial secretions	ICU	31 August 2023	*bla_KPC_* + *bla_NDM_*	Colistin, Tigecycline
D4784	bronchial secretions	ICU	19 September 2023	*bla_KPC_*	Ceftazidime/avibactam, Meropenem/vaborbactam, Imipenem/relebactam
D5316	bronchial secretions	ICU	18 October 2023	*bla_NDM_*	Tigecycline
E787	central venous catheter	ICU	4 June 2023	*bla_KPC_*, *bla_NDM_*	Resistant to all tested antibiotics
E1119	central venous catheter	ICU	9 August 2023	*bla_KPC_*, *bla_NDM_*	Colistin, Tigecycline
E1217	central venous catheter	ICU	30 August 2023	*bla_KPC_*, *bla_NDM_*	Tigecycline (I)
E1395	central venous catheter	ICU	8 October 2023	*bla_KPC_*, *bla_NDM_*	Tigecycline
E1403	central venous catheter	ICU	10 October 2023	*bla_NDM_*	Tigecycline (I)

**Table 2:** Antimicrobial susceptibility profile of 32 carbapenem-resistant *K. pneumonia* isolates. All intermediate susceptibility results were grouped with the susceptible. Abbreviations: I: Susceptible, increased exposure (Eucast), I: Intermediate (F.D.A), *n*: number of isolates.

Antibiotic	MIC Breakpoints (≤S/>R)	Sensitive (S) *n*	Susceptible, Increased Exposure (Eucast) Intermediate (I) *n*	Resistant^®^ *n*
**β-lactams**				
Ampicillin	≤8/>8	0	0	32
Piperacillin/tazobactam	≤8/>8	0	0	32
Ceftazidime/avibactam	≤8/>8	8	0	24
Meropenem/vaborbactam	≤8/>8	8	0	24
Meropenem	≤2/>8	0	0	32
Ertapenem	≤0.5/>0.5	0	0	32
Imipenem/relebactam	≤2/>2	8	0	24
Imipenem	≤2/>4	0	0	32
Ceftolozane/tazobactam	≤2/>2	0	0	32
Cefepime	≤1/>4	0	0	32
Ceftazidime	≤1/>4	0	0	32
Ceftriaxone	≤1/>2	0	0	32
Cefotaxime	≤1/>2	0	0	32
Aztreonam	≤1/>4	1	1	30
**Aminoglycosides**				
Gentamicin	≤2/>2	9	0	23
Tobramycin	≤2/>2	0	0	23
Amikacin	≤8/>8	0	0	23
**Fluoroquinolones**				
Ciprofloxacin	≤0.25/>0.5	0	0	32
Levofloxacin	≤0.5/>1	1	0	31
**Tetracyclines**				
Tigecycline	≤2/>8	21	7	4
**Miscellaneous agents**				
Fosfomycin	≤32/>32	0	0	32
Trimethoprim/sulfamethoxazole	≤40/>80	4	0	28
Colistin	≤2/>2	12	0	20

**Table 3:** MICs (μg/L) of 16 antibiotics for the 32 strains of the study.

≥ID	Carbapenemases	Imipenem ≤2/>4	Imipenem–Relebactam ≤2/>2	Meropenem ≤2/>8	Meropenem-Vaborbarctam ≤8/>8	Piperacillin–Tazobactam ≤8/>8	Ceftazidime ≤1/>4	Ceftazidime–Avibactam ≤8/>8	Aztreonam ≤1/>4	Ceftriaxone ≤1/>2	Cefepime ≤1/>4	Amikacin ≤8/>8	Gentamycin ≤2/>2	Ciprofloxacin ≤0.25/>0.5	Levofloxacin ≤0.5/>1	Tigecycline ≤2/>8	Colistin ≤2/>2	Trimethoprim–Sulfamethoxazole ≤40/>80
A9072	KPC	≥16	0.5	≥16	0.5	≥128	≥64	4	≥64	≥64	≥32	≥32	2	≥4	≥8	1	4	≥320
A11159	KPC	≥16	0.5	≥16	0.5	≥128	32	1	≥64	≥64	≥32	≥32	≤1	≥4	4	≤0.5	0.25	≤20
A11694	NDM	≥16	≥16	≥16	≥64	≥128	≥64	≥16	≥64	≥64	≥32	≥32	≥16	≥4	≥8	2	16	≥320
A15330	KPC	≥16	0.5	≥16	0.5	≥128	≥64	1	≥64	≥64	≥32	≥32	≤1	≥4	≥8	4	16	≤20
A18251	KPC,NDM	≥16	≥16	≥16	≥64	≥128	≥64	≥16	≥64	≥64	≥64	≥32	≥16	≥4	≥8	4	16	≥320
A20170	NDM	≥16	≥16	≥16	32	≥128	≥64	≥16	≥64	≥64	≥32	≥32	≥16	≥4	≥8	≥8	0.25	≥320
B7232	NDM	≥16	≥16	≥16	32	≥128	≥64	≥16	≥64	≥64	≥32	≥32	≥16	≥4	≥8	≥8	1	≥320
B9217	NDM	8	≥16	≥16	32	≥128	≥64	≥16	≥64	≥64	≥32	≥32	≥16	≥4	≥8	1	0.25	≤20
B12743	NDM	≥16	≥16	≥16	≥64	≥128	≥64	≥16	2	≥64	≥32	≥32	≥16	≥4	1	4	16	≥320
B13435	NDM,KPC	≥16	≥16	≥16	≥64	≥128	≥64	≥16	≥64	≥64	≥32	≥32	≥16	≥4	≥8	1	16	≥320
C4171	NDM	≥16	≥16	≥16	32	≥128	≥64	≥16	≤1	≥64	≥32	≥32	≤1	≥4	≥8	2	8	≥320
C5596	KPC	≥16	0.5	≥16	0.5	≥128	≥64	1	≥64	≥64	≥32	≥32	≤1	≥4	≥8	4	4	≥320
C6168-1	NDM,KPC	≥16	≥16	≥16	≥64	≥128	≥64	≥16	≥64	≥64	≥32	≥32	≥16	≥4	≥8	1	16	≥320
D2415	NDM,KPC	≥16	≥16	≥16	16	≥128	≥64	≥16	≥64	≥64	≥32	≥32	≥16	≥4	≥8	1	0.5	≥320
D2606	KPC	8	0.5	≥16	0.5	≥128	≥64	0.5	≥64	≥64	≥32	≥32	≤1	≥4	≥8	1	0.25	≥320
D2678	NDM,KPC	≥16	≥16	≥16	32	≥128	≥64	≥16	≥64	≥64	≥32	≥32	≤1	≥4	≥8	1	1	≥320
D2803	NDM,KPC	≥16	≥16	≥16	≥64	≥128	≥64	≥16	≥64	≥64	≥32	≥32	≥16	≥4	≥8	1	1	≥320
D2845	NDM,KPC	≥16	≥16	≥16	16	≥128	≥64	≥16	≥64	≥64	≥32	≥32	≥16	≥4	≥8	1	16	≥320
D3437-2	KPC	≥16	0.5	≥16	0.5	≥128	32	1	≥64	≥64	≥32	≥32	2	≥4	≥8	≤0.5	0.25	≤20
D4109	NDM,KPC	≥16	≥16	≥16	≥64	≥128	≥64	≥16	≥64	≥64	≥32	≥32	≥16	≥4	≥8	2	16	≥320
D4140	KPC	≥16	0.5	≥16	0.5	≥128	32	1	≥64	≥64	≥32	≥32	≤1	≥4	≥8	4	16	≤20
D4175	NDM,KPC	≥16	≥16	≥16	32	≥128	≥64	≥16	≥64	≥64	≥32	≥32	≥16	≥4	≥8	1	16	≥320
D4370	NDM,KPC	≥16	≥16	≥16	≥64	≥128	≥64	≥16	≥64	≥64	≥32	≥32	≥16	≥4	≥8	1	2	≥320
D4449	NDM,KPC	≥16	≥16	≥16	16	≥128	≥64	≥16	≥64	≥64	≥32	≥32	≥16	≥4	≥8	2	8	≥320
D4459	NDM,KPC	≥16	≥16	≥16	32	≥128	≥64	≥16	≥64	≥64	≥32	≥32	≥16	≥4	≥8	2	1	≥320
D4784	KPC	≥16	≤0.25	≥16	0.5	≥128	32	0.5	≥64	≥64	32	≥32	8	1	2	≥8	8	160
D5316	NDM	≥16	≥16	≥16	32	≥128	≥64	≥16	≥64	≥64	≥32	≥32	≥16	≥4	≥8	1	16	≥320
E787	NDM,KPC	≥16	≥16	≥16	32	≥128	≥64	≥16	≥64	≥64	≥32	≥32	≥16	≥4	≥8	≥8	16	≥320
E1119	NDM,KPC	≥16		≥16	≥64	≥128	≥64	≥16	≥64	≥64	≥32	≥32	≥16	≥4	≥8	1	1	≥320
E1217	NDM,KPC	≥16	≥16	≥16	64	≥128	≥64	≥16	≥64	≥64	≥32	≥32	≥16	≥4	≥8	4	16	≥320
E1395	NDM,KPC	≥16	≥16	≥16	16	≥28	≥64	≥16	≥64	≥64	≥32	≥32	≥16	≥4	≥8	1	16	≥320
E1403	NDM	≥16	≥16	≥16	32	≥128	≥64	≥16	≥64	≥64	≥32	≥32	≥16	≥4	≥8	4	16	≥320

**Table 4:** Key molecular findings of WGS of isolate D4370.

Isolate ID	ST/Clonal Group	Carbapenemase Genes	Other β-Lactamase Genes	Aminoglycoside Resistance	Fluoroquinolone Resistance	Other Resistance Genes	Key Mutations	Plasmid Replicons	Virulence Factors
D4370 (GRTHES)	ST512(CG258)	*bla*_NDM-1_, *bla*_KPC-3_	*bla*TEM-1, *bla*OXA-1, *bla*SHV-11, *bla*CTX-M-15	*aph*(3″)-Ib, *aph*(6)-Id, *aac*(3)-IIe, *aac*(6′)-Ib, *aad*A2	*oqx*A, *oqx*B	*sul*1, *sul*2, *cat*A1, *cat*B3, *dfr*A12, *dfr*A14, *ble*, *mph*(A), *qac*EΔ1, *fos*A	*par*C S80I, *gyr*A S83I (fluoroquinolones); *mgr*B G37S, *pmr*B R256G (colistin); *omp*K36 D135DGD (carbapenems)	IncFIA(HI1), IncFIB(K), IncFII(K), IncX3	yersiniabactin (*ybt*, *fyu*A), enterobactin (*ent*A, *ent*B, *fep*C), *ecp*ABCDE, *omp*A

**Table 5:** Primers used for multiplex PCR.

Target Gene	Primer Name	Sequence (5′→3′)	Product Size (bp)	Reference
*bla*_NDM_	NDM-F	GGTTTGGCGATCTGGTTTTC	621	Ellington et al. [39], Poirel et al. [40]
	NDM-R	CGGAATGGCTCATCACGATC		
*bla*_VIM_	VIM-F	GATGGTGTTTGGTCGCATA	390	Ellington et al. [39], Poirel et al. [40]
	VIM-R	CGAATGCGCAGCACCAG		
*bla*_OXA-48_	OXA-F	GCGTGGTTAAGGATGAACAC	438	Ellington et al. [39], Poirel et al. [40]
	OXA-R	CATCAAGTTCAACCCAACCG		
*bla*_KPC_	KPC-Fm	CGTCTAGTTCTGCTGTCTTG	798	Ellington et al. [39], Poirel et al. [40]
	KPC-Rm	CTTGTCATCCTTGTTAGGCG		

## Data Availability

The whole genome of GRTHES *K. pneumoniae* has been deposited at DDBJ/ENA/GenBank under the accession Number JBJFLW000000000.
